# Predicting PBT and CMR properties of substances of very high concern (SVHCs) using QSAR models, and application for K-REACH

**DOI:** 10.1016/j.toxrep.2020.08.014

**Published:** 2020-08-15

**Authors:** Joonsik Moon, Byongcheun Lee, Jin-Sung Ra, Ki-Tae Kim

**Affiliations:** a Department of Environmental Energy Engineering, Seoul National University of Science and Technology, Seoul, 01811, Republic of Korea; b Risk Assessment Division, National Institute of Environmental Research, Incheon, 22689, Republic of Korea; c Eco-testing and Risk Assessment Center, Korea Institute of Industrial Technology (KITECH), Ansan, 15588, Republic of Korea

**Keywords:** AD, applicability domain, AFC, atom/fragment contribution, BCF, bioconcentration factor, CAESAR, Computer Assisted Evaluation of industrial chemical Substances According to Regulations, CAS, chemicals abstracts service, CMR, carcinogenic, mutagenic or toxic for reproduction, DSSTox, distributed structure-searchable toxicity, ECHA, European Chemical Agency, EDC, endocrine disrupting chemicals, EPI, estimation programs interface, FN, false negative, FP, false positive, GHS, globally harmonized system of classification and labelling of chemicals, Kow, octanol-water coefficient, LAZAR, lazy structure–activity relationships, PBT, persistent, bioaccumulative and toxic, PFCAs, perfluorinated carboxylic acids, PFDA, nonadecafluorodecanoic acid, QMRF, QSAR model reporting format, QPRF, QSAR prediction reporting format, QSAR, quantitative structure-activity relationship, REACH, registration, evaluation, authorization and restriction of chemicals, SA, structure alters, SMILES, simplified molecular-input line-entry system, SVHCs, substances of very high concern, TN, ture negative, TP, ture positive, US EPA, United States Environmental Protection Agency, UVCBs, complex reaction products or biological materials, WoE, weight of evidence, QSAR, SVHCs, PBT, CMR, K-REACH

## Abstract

Quantitative structure-activity relationship (QSAR) models have been applied to predict a variety of toxicity endpoints. Their performance needs to be validated, in a variety of cases, to increase their applicability to chemical regulation. Using the data set of substances of very high concern (SVHCs), the performance of QSAR models were evaluated to predict the persistence and bioaccumulation of PBT, and the carcinogenicity and mutagenicity of CMR. BIOWIN and Toxtree showed higher sensitivity than other QSAR models – the former for persistence and bioaccumulation, the latter for carcinogenicity. In terms of mutagenicity, the sensitivities of QSAR models were underestimated, Toxtree was more accurate and specific than lazy structure–activity relationships (LAZARs) and Computer Assisted Evaluation of industrial chemical Substances According to Regulations (CAESAR). Using the weight of evidence (WoE) approach, which integrates results of individual QSAR models, enhanced the sensitivity of each toxicity endpoint. On the basis of obtained results, in particular the prediction of persistence and bioaccumulation by KOWWIN, a conservative criterion is recommended of log Kow greater than 4.5 in K-REACH, without an upper limit. This study suggests that reliable production of toxicity data by QSAR models is facilitated by a better understanding of the performance of these models.

## Highlights

• BIOWIN is effective for predicting persistence and bioaccumulation.
• Toxtree is effective for predicting carcinogenicity and mutagenicity.
• WoE approach enhances the sensitivity.
• It is recommended to set a conservative criteria of log Kow more than 4.5 in K-REACH.

## 1 Introduction

SVHCs regulated by REACH are chemicals which include substances that are PBT and CMR substances. The manufacturers and importers of SVHCs are subject to authorization for use or distribution under the REACH regulation. Between 2008 and 2018, the ECHA published a candidate list of 191 chemicals or chemical groups as SVHCs requiring authorization, which has constantly been updated [1]. However, experimental data produced using animal models are accompanied by several limitations such as the amount of time and money required, and ethical issues [2]. Effective non-animal testing methods are required for assessment of a various class of chemicals for which the hazard is unknown.

The computational toxicology, *in silico*, has been suggested as a means of screening for chemical toxicity and determining the chemicals to be prioritized for further toxicity testing [3]. QSAR models used in one type of computational method have been widely accepted as able to predict a variety of toxicity endpoints on the basis of their structural properties. QSAR models exhibit the mathematically quantitative relationship between physicochemical properties or molecular descriptors and biological activity in a certain data set. Notable institutions managing domestic or international chemicals, such as the OECD, ECHA and the US EPA, have embraced the use of QSAR models and published related guidelines [[4], [5], [6]]. The OECD announced five principles to consider when using QSAR models for defining an unambiguous endpoint and algorithm, applying a defined domain, interpreting a mechanism, and measuring goodness-of-fit and predictability [4]. In a recent survey study, toxicological experts and the general users expressed optimistic views on application of QSAR models for toxicity screening assessment [7]. Previous studies have proven the performance of QSAR models for different toxicity endpoints, depending on their respective data sets. Milan et al. [8] and NIER [9] evaluated QSAR models regarding carcinogenicity by comparing predicted and measured data of 1,500 organic chemicals. Guerra et al. [2] validated three QSAR models regarding carcinogenicity and mutagenicity using 37 volatile organic compounds (VOCs) as the targeted data set. Cassano et al. [10] and NIER [11] employed multiple QSAR models to evaluate the genotoxicity of organic chemicals. It is believed that more case studies could result in expanded application of QSAR models [12].

We first aimed to examine the performance of QSAR models by predicting PBT and CMR properties used to identify SVHCs and employed those QSAR models recommended by Korean REACH (“K-REACH”). In PBT, the term toxicity (T) was excluded because it covers a wide range of unspecified adverse effects classified in GHS, which was inappropriate to evaluate performance of specific QSAR models. In addition, in CMR, applying the WoE approach is impossible because among QSAR model candidates, only CAESAR is available for predicting reproductive toxicity (R). Thus, reproductive toxicity was not considered. Therefore, we selected QSAR models to predict the persistence and bioaccumulation of PBT, and the carcinogenicity and mutagenicity of CMR. The selected models were KOWWIN, BIOWIN, BCFBAF and HYDROWIN for persistence and bioaccumulation, and Toxtree, LAZAR and CAESAR for carcinogenicity and mutagenicity. The candidate list of SVHCs for authorization was used as the data set. The list of SVHCs is suitable to evaluate the performance of QSAR models in that it includes organic, inorganic and metallic compounds and is recognized as reliable for the examination process by using available data.

The second objective of this study was to integrate the predicted results from the individual QSAR models and evaluate this weighted performance. The WoE approach was applied to the chemical assessments as integral data of several independent information to demonstrate its reliability in consideration of the performance of QSAR models.

## 2 Materials and methods

### 2.1 QSAR models on persistence and bioaccumulation

KOWWIN, BIOWIN, BCFBAF and HYDROWIN are included in the EPI Suite (V. 4.11) provided by the US EPA. These QSAR models were developed and proposed to estimate the persistence and bioaccumulation of organic chemicals using direct or indirect factors such as Kow, the half-life of hydrolysis and BCF. The SMILES of targeted chemical is required for QSAR model input data, and the CAS registry number is also used.

A summary of the prediction principles for each QSAR model follows. KOWWIN predicts the log Kow values using the AFC method dividing the entire chemical structure into individual fragments, with the Kow of fragments added up. The log Kow value of each fragment is derived by multiple regression analysis from a total of 2,447 measured values. BIOWIN predicts the biodegradation period of the chemical and calculates BIOWIN 1–7 according to the different analysis methods and type of biodegradation (aerobic/anaerobic). BCFBAF calculates the BCF of target chemicals by classifying them as ionic or non-ionic. Carboxylic acid, sulfonic acid and its salts and charged nitrogen compounds are classified as ionic chemicals and other chemicals are classified as non-ionic. In addition, the log Kow range of a chemical is considered as an important factor in BCF prediction. HYDROWIN predicts the half-life of chemicals that can be hydrolyzed in water, which includes alkyl halides, carbamate, epoxides, esters, halomethanes, and phosphorus esters, as they are vulnerable to hydrolysis.

In this study, the predicted results from QSAR models were applied to the criteria provided by K-REACH as shown in Table 1. Through this process, all results were reconstructed into persistent/bioaccumulative (positive) or not persistent/bioaccumulative (negative).

**Table 1:** Evaluation criteria for persistence and bioaccumulation using QSAR [13].

QSAR model	Type of result	Criteria
KOWWIN	Octanol-Water partition coefficient (K_ow_)	4.7 <log K_ow_< 7.6
BCFBAF	Bioconcentration factor (BCF)	1000 ≤ BCF
BIOWIN	BIOWIN 2, 3, 6	BIOWIN 2, 6: Not biodegradable BIOWIN 3 ≥ month
HYDROWIN	Half-life of hydrolysis (days)	> 14 days

### 2.2 QSAR models on carcinogenicity and mutagenicity

Toxtree (V.3.1.0), LAZAR (V.1.4.2), and CAESAR (V.2.1.9 for carcinogenicity and V.2.1.13 for mutagenicity) were used to predict the carcinogenicity and mutagenicity of SVHCs. SMILES is used as the chemical input information in three QSAR models, and the CAS number is, in part, used in Toxtree. In order to predict the carcinogenicity and mutagenicity, the rule base method is applied to Toxtree, while the database method is applied in LAZAR and CAESAR [8]. The rules provided in Toxtree are the sequential algorithms composed by the SAs that cause carcinogenic or mutagenic effects, while the results of Toxtree depend on whether the SAs are identified in the targeted chemical. The 'Benigni/Bossa rule base for mutagenicity and carcinogenicity' was chosen from 18 plugins covering a wide range of toxicity endpoints in Toxtree and used to identify carcinogens, while ‘in vitro mutagenicity (Ames test) alerts by ISS’ was used for mutants. On the other hand, a database that includes test data for carcinogenic and mutagenic chemicals is built into LAZAR and CAESAR. Carcinogenicity and mutagenicity are predicted by test data of the analogues structurally similar to the targeted chemical in the database. The LAZAR model is a data mining method that uses a training set instead of chemical and biological information to predict toxicity endpoints. The CAESAR (from VEGAHUB) carcinogenicity model consists of a Counter Propagation Artificial Neural Network (CP ANN), which holds 12 descriptors. Mutation prediction uses a combination of Support Vector Machine (SVM) classifier and a model for SA matching.

In this study, carcinogenicity as determined by Toxtree was deemed negative only if the predicted results were negative for both genotoxic and nongenotoxic carcinogenicity. In LAZAR, three predicted results for rat, mouse and rodent animal models were offered in carcinogenicity. If at least one of the three groups were positive, carcinogenicity was determined as positive. In the predicted result displayed in CAESAR, red was regarded as positive, green as negative. On the other hand, mutagenicity was determined without any other process, as all QSAR models predicted only one result for the Ames test for salmonella typhimurium.

### 2.3 Analysis method of QSAR model validation

The validation of QSAR models was performed according to statistical method found in Cooper et al. [14]. The compared results were sorted according to a matrix consisting of TP, FP, TN, and FN. Performance was quantified by calculating accuracy, sensitivity and specificity. Accuracy is defined as (TP + TN) / (FP + TP + FN + TN), Sensitivity isTP / (TP + FN), and specificity is TN / (TN + FP), respectively. Accuracy is the inverse proportion to the ratio predicted to FP and FN. Therefore, high accuracy means a low number of false results predicted in QSAR models. Sensitivity is the inverse proportion to the ratio predicted to FN, while specificity is the inverse proportion to the ratio predicted to FP. These factors represent the level of performance of the QSAR models. Of the above terms, sensitivity can be accepted as the most important in chemical regulation. From a conservative viewpoint, chemical regulation should previse the risk of harm from the use of hazardous chemicals, while un-regulated chemicals must be safe to use.

### 2.4 Weight of evidence in QSAR model

The WoE approach was determined by selecting the high frequency results that were predicted in individual QSAR models. For example, if there were at least two positive values of persistence and bioaccumulation predicted in KOWWIN, BIOWIN, BCFBAF and HYDROWIN, a positive value was chosen as a result of WoE. Similarly, for carcinogenicity and mutagenicity, a positive value was chosen as a result of WoE if at least two positive values were predicted in Toxtree, LAZAR and CAESAR. WoE was validated in the same way as for individual QSAR models.

## 3 Results

### 3.1 Data set for validation

In the list of SVHCs, the chemicals that are not suitable for the QSAR models were identified. For example, there are groups of chemicals like PFDA and other formula (i.e., its sodium and ammonium salts), or those of UVCBs like anthracene oil. Firstly, the chemicals in a certain group were evaluated as duplicates due to their identical or similar structures that can be expected to yield the same results, which might exaggerate or reduce the performance of a QSAR model. Secondly, QSAR models are not suitable for UVCBs because their SMILES cannot be determined. In this study, only one representative chemical included in each group was considered in our data set, and UVCBs were excluded. As a result, a final data set of 179 chemicals was decided. Carcinogenicity and mutagenicity were predicted in a total of 179 SVHCs, but only 108 organic chemicals were selected for persistence and bioaccumulation, while 71 inorganic, ionic, and metallic chemicals were excluded.

Through KOWWIN, BIOWIN and BCFBAF, all 108 organic SVHCs were predicted, however, only 32 chemicals were predicted through HYDROWIN. As previously noted, HYDROWIN was developed for use with chemicals that can be hydrolyzed. Therefore, the remaining 76 unpredicted organic SVHCs in HYDROWIN are not hydrolysable chemicals. All 179 SVHCs were predicted for carcinogenicity and mutagenicity in Toxtree. However, several metallic SVHCs such as cadmium, lead and chromium compounds were unpredicted in LAZAR and CAESAR (Table 2).

**Table 2:** Number of SVHCs predicted in QSAR models.

QSAR model	Endpoint	Type of chemical	Number (%)
KOWWIN	Persistence/bioaccumulation	Organic chemicals (108)	108 (100)
BIOWIN			108 (100)
BCFBAF			108 (100)
HYDROWIN			32 (30)
Toxtree	Carcinogenicity	All chemicals (179)	179 (100)
	Mutagenicity		179 (100)
LAZAR	Carcinogenicity		139 (78)
	Mutagenicity		119 (66)
CAESAR	Carcinogenicity		136 (76)
	Mutagenicity		123 (69)

### 3.2 Performance of QSAR models and WoE in persistence and bioaccumulation

**Fig. 1:**
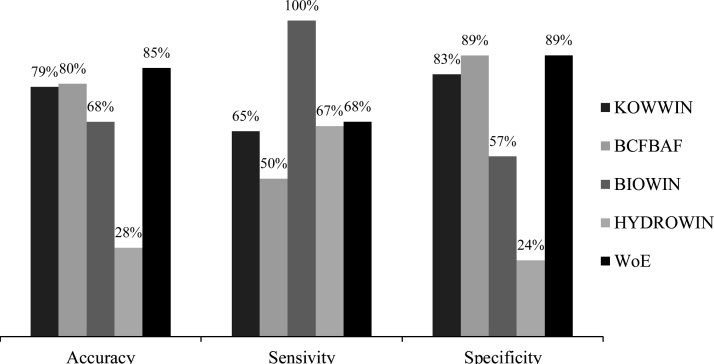
Performances of KOWWIN, BCFBAF, BIOWIN, HYDROWIN and WoE in persistence and bioaccumulation.

BCFBAF exhibited the highest accuracy (80 %) and specificity (89 %), but sensitivity was highest in BIOWIN (100 %). KOWWIN showed moderate performance similar to BCFBAF and BIOWIN. However, KOWWIN had accuracy, sensitivity and specificity rates of only 28 %, 67 % and 24 %, respectively - lower than other QSAR models. For WoE, sensitivity was higher than individual QSAR models except BIOWIN, while accuracy was higher than all of the QSAR models (Fig. 1).

### 3.3 Performance of QSAR models and WoE in carcinogenicity and mutagenicity

**Fig. 2:**
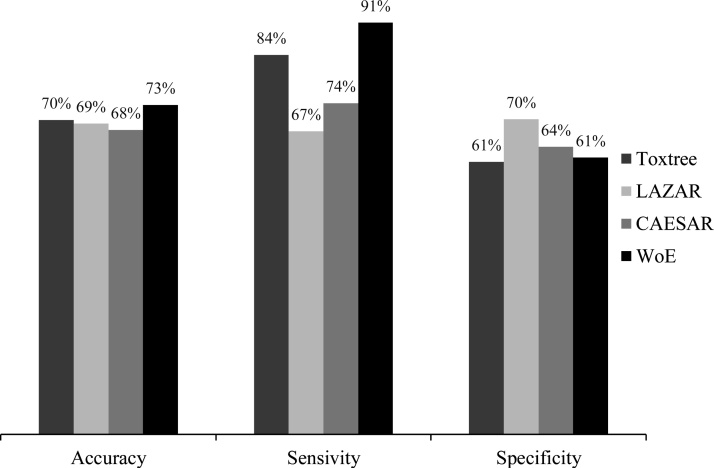
Performances of Toxtree, LAZAR, CAESAR and WoE in carcinogenicity.

In terms of carcinogenicity, Toxtree had 70 % accuracy, 84 % sensitivity, and 61 % specificity. Toxtree had the highest accuracy and sensitivity of the QSAR models. However, LAZAR and CAESAR were higher than Toxtree in specificity. WoE was higher than each of the other QSAR models in accuracy (73 %) and sensitivity (91 %). WoE had 61 % specificity, which was higher than Toxtree but lower than LAZAR and CAESAR (Fig. 2).

**Fig. 3:**
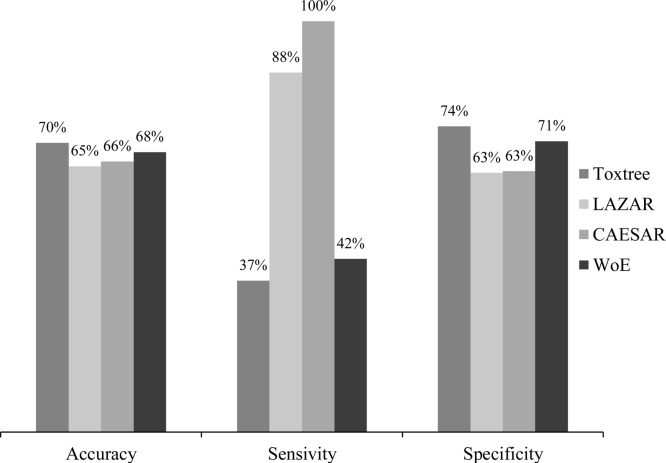
Performances of Toxtree, LAZAR, CAESAR and WoE in mutagenicity.

Toxtree, LAZAR and CAESAR showed similar accuracy and specificity in mutagenicity. However, the sensitivity of LAZAR (89 %) and CAESAR (100 %) were much higher than Toxtree’s 37 % (Fig. 3). WoE showed 68 % accuracy, 42 % sensitivity and 71 % specificity.

## 4 Discussion

We evaluated QSAR model performance in terms of accuracy, sensitivity and specificity defined above. In persistence and bioaccumulation, the QSAR models with high accuracy were in descending order of BCFBAF, KOWWIN and BIOWIN. The tendency between sensitivity and specificity were opposite in BIOWIN and BCFBAF. BIOWIN was the highest for sensitivity and lowest for specificity while BCFBAF was the highest for specificity and lowest for sensitivity. On the other hand, the performance of HYDROWIN was more limited than other QSAR models, and fewer chemicals were evaluated, leaving out 76 of 108 hydrolysable substances. In terms of carcinogenicity, Toxtree was the highest for accuracy and sensitivity but the lowest for specificity. LAZAR was the highest for specificity but the lowest for sensitivity. Interestingly, these tendencies of the QSAR models obtained in this study were different from other studies [2,8]. Toxtree showed higher specificity and lower sensitivity than LAZAR in Guerra et al. [2], while CAESAR showed higher accuracy, sensitivity and specificity than Toxtree and LAZAR. These differences resulted from difference in data sets - in other words, the varying characteristics of targeted chemicals. Guerra et al. [2] used VOCs and Milan et al. [8] used organic chemicals for data sets, including many chemicals from the DSSTox database, which is a database built into CAESAR. It has been reported that diverse differences in performance of QSAR models were dependent on type and size of data set [15,16]. The performance of QSAR models can be enhanced through the inclusion of more chemicals and it is also affected how chemicals used to training set in QSAR models. Milan et al. [8] evaluated the performance in two cases by classifying chemicals into ‘in training set’ (in DB) and ‘test set’ (out DB), and reported that performance of in DB was higher than out DB. It is possible that chemicals tested in this study were included in the database of each QSAR model. In addition, predictive reliability can be improved if several models are used. We applied CAESAR (from VEGAHUB) to predict carcinogenicity. However, more accurate results can be obtained by applying all models in VEGAHUB as opposed to only using CAESAR.

The sensitivity of the QSAR model is the most relevant performance factor to conservative concepts pursued in chemical regulations [10,17]. When users select a QSAR model for regulatory purposes, BIOWIN is recommended as suitable for persistence and bioaccumulation, while Toxtree is suitable for carcinogenicity, because they showed higher sensitivity than other QSAR models. However, even though they perform well, it is difficult to ensure QSAR model reliability because of the complexity of toxicity endpoints, uncertainties in the database, and the presence of unidentified toxic fragments [18,19]. The AD, the information or knowledge obtained from the training set used to develop the QSAR model, is an important factor when users seek to predict the toxicity of chemicals using a QSAR model. Such prediction in new chemicals should be carried out within the AD, and chemical prediction outside the AD should be avoided [20,21]. From the findings of this study, the chemicals classified as FN were identified, indicating that the chemicals were out of AD and resulted in weak sensitivity in a QSAR model. For example, perfluorinated carboxylic acids (PFCAs) were repeatedly identified as FN chemicals in QSAR models in predicting persistence and bioaccumulation. PFCAs are indeed persistent and bioaccumulative but their log Kow is low in the experiment, because PFCAs easily form an ionic structure. Therefore, the QSAR models related to log Kow are not recommended for evaluating persistence and bioaccumulation of chemicals whose physicochemical properties cannot reflect a realistic chemical form like PFOAs [22,23]. Regarding carcinogenicity, several metal compounds were identified as FN in the QSAR models. In Toxtree, nine carcinogenic cadmium compounds were predicted to be non-carcinogenic. In LAZAR and CAESAR, numbers of metal compounds including cadmium, chromium, cobalt or lead were also classified as FN or not predicted. The predictive reliability of QSAR models used in this study appears to be limited to organic chemicals, and inaccurate with inorganic and metallic compounds. We speculate that toxic fragments have yet to be sufficiently identified, and the database and prediction methods need to be updated.

In terms of mutagenicity, Toxtree showed a much lower sensitivity than LAZAR and CAESAR – contrary to the case with carcinogenicity. However, we found that the data set included 19 mutagenic chemicals, which were mostly inorganic or metallic compounds. As identified in the carcinogenicity, LAZAR and CAESAR were inaccurate to predict inorganic and metallic compounds. As a result, most mutagenic chemicals in the data set were not predicted by LAZAR and CAESAR and were not involved in the calculation of sensitivity. We speculate that the imbalance of results on positive and negative data, which was also observed in BCFBAF prediction, could influence the behavior of Cooper’s statistics. Therefore, the sensitivity of QSAR models to mutagenicity would seem to be underestimated. Also, it is possible that the Ames test does not guarantee the mutagenic. For instance, 4-aminoazobenzene is labelled as false positive for mutagenicity in this study although it is mutagenic according to the Ames test. Given that the Ames test is an alternative screening test for mutagenicity, it is practical that QSAR models based on it can be used to predict mutagenecity.

In this study, WoE approaches were conducted in terms of the frequency of results predicted from individual QSAR models. The employment of WoE approaches mostly enhanced accuracy in persistence, bioaccumulation and carcinogenicity when compared to reliance on individual QSAR models. And, the specificity by WoE was similar or slightly lower than that of individual QSAR models. However, in terms of sensitivity, the resulting performance varied according to toxicity endpoints. In persistence and bioaccumulation, BIOWIN was higher than WoE, whilst in mutagenicity performance was higher than the WoE approach when applying individual LAZAR and CAESAR. The performance tendency of WoE varies depending on how results predicted from various models were combined. For persistence and bioaccumulation, the performance of WoE was lower than expected because of the methodology of combination. In combined QSAR models, the previous studies have reported that the performance of a WoE approach was affected by the methodology of combination, the type of toxicity endpoint, and the selection of individual QSAR models [8,24,25]. Orogo et al. [24] combined the predicted results of three QSAR models using two methods. The first method was very conservative in that if one individual QSAR model predicted a positive value, the WoE approach determined a positive outcome. The second method determined the high frequency value among the predicted values in the individual QSAR models. Between these two methods, we can expect that the first method increased the sensitivity of the WoE approach than the second method would. As another case study, Nendza et al. [25] combined the predicted results of 7 QSAR models for persistence and bioaccumulation, similar to the first method utilized by Orogo et al. [24], resulting in 100 % sensitivity in the WoE approach. Milan et al. [8] also demonstrated an increase in sensitivity by combining models for carcinogenicity. For mutagenicity, we speculate that the performance of the WoE approach is low because a number of chemicals out of AD were assessed. Similar results were found in Cassano et al. [10], who conducted a WoE approach by dividing two groups for the chemical of target. The chemicals in the first group were involved in the AD in all QSAR models, and chemicals in the second group were not. This resulted in the performance of the WoE approach in the first group being higher than that of the second group. Therefore, by combing individual results, the involvement of targeted chemicals in AD should be taken into consideration to increase the efficiency of WoE approaches. Collectively, the WoE approach can be viewed as a reliable way beyond individual QSAR models, but it should be noted that performance is determined by various factors such as selecting the appropriate QSAR model according to the endpoints, including the chemical to be predicted in the AD, and determining the methodology for the WoE approach. For example, although an objective standard is required, QSAR models can be sequentially applied. For carcinogenicity, the rule base Toxtree can be first considered, and LAZAR, which provides a similar approach with read-across as a non-parametric tool, can be considered last. Further research on various WoE methods is needed to integrate individual results of QSAR models and to obtain better predictive reliability from them.

### 4.1 Implication for K-REACH

Inspired by EU REACH, K-REACH has been enforced since 2015 to regulate chemical substance and products in the Republic of Korea. Under K-REACH, SVHCs have been managed since 2019 under ‘Key Management Substances’. On the list of key management substances, 204 chemicals exhibit the properties of CMR or PBT. K-REACH recommends utilizing alternative testing methods and using existing data to follow the principle of minimizing animal testing. If the amount of a specific chemical imported or manufactured is less than ten tons per year, QSAR data can produce four human hazards and three environmental hazards. The submitter of the QSAR data is required to use the QMRF and QPRF in their reports. QSAR models can also be used in the toxicity data required in the CSR, as they provide indirect evidence for exemptions, supplementation and reduction of uncertainty [26]. Log Kow is an important indicator for bioaccumulation and is used for screening PBT materials. It is also widely accepted that the chemicals with high log Kow are not accumulate in the body [27]. Given the guidelines for application of QSAR models in K-REACH, the criteria for chemical persistence and bioaccumulation are log Kow in the range of 7.6 > log Kow> 4.5. However, in the prediction of this study using KOWWIN, some chemicals greater than log Kow of 7.6 identified as PBT substances. It is possible that Kow values can be mis-calcualted by KOWWIN in which they are estimated by the summation of Kow values in each fragment. This can mislead researchers regarding actual Kow values based on intrisic physicochemical properties. Therefore, it seems that persistence and bioaccumulation are underestimated when using the upper limit of 7.6 in that the objective for using QSAR models is to identify the characteristics of chemicals without further testing.

In EU REACH, the range of log Kow is just over 4.5. Therefore, it is recommended to set a conservative range of criteria for log Kow in K-REACH. In addition, the manufacture or import of at least 100 kg/year of non-phase in substances or at least one ton per year of phase-in substances shall be ‘registered’ according to K-REACH. Although K-REACH allows a grace period depending on toxicity or tonnage, all substances must be registered within 10 years. Therefore, further use of alternative QSAR models is expected to grow and practical measures will be required. The expansion of AD is necessary, and reliable QSAR models should be developed to predict several toxicity endpoints of inorganic and metallic compounds.

## 5 Conclusion

The performance of QSAR models using a SVHC data set were examined. We evaluated KOWWIN, BIOWIN, BCFBAF and HYDROWIN in terms of persistence and bioaccumulation, and Toxtree, LAZAR and CAESAR in terms of carcinogenicity and mutagenicity. In terms of regulatory purpose, BIOWIN showed higher sensitivity to persistence and bioaccumulation and Toxtree for carcinogenicity of the other QSAR models. In terms of mutagenicity, the sensitivities of QSAR models were underestimated due to a lower number of mutagenic chemicals in the data set, but Toxtree is effective in terms of accuracy and specificity. The WoE approach, which integrates results of individual QSAR models, showed enhanced sensitivity. We validated QSAR performance by using a SVHC data set and suggested that a better understanding of their performance promotes the application of toxicity data they produce into alternative methods of chemical regulation.

## CRediT authorship contribution statement

**Joonsik Moon:** Writing - original draft, Investigation, Writing - review & editing. **Byongcheun Lee:** Resources, Writing - review & editing. **Jin-Sung Ra:** Resources, Writing - review & editing. **Ki-Tae Kim:** Supervision, Project administration, Writing - review & editing.

## Declaration of Competing Interest

The authors declare that they have no known competing financial interests or personal relationships that could have appeared to influence the work reported in this paper.
