# Supplementary material for: Predicting PBT and CMR properties of substances of very high concern (SVHCs) using QSAR models, and application for K-REACH
Source: Toxicol Rep. 2020 Aug 15;7:995–1000. doi: 10.1016/j.toxrep.2020.08.014 (PMC7451722; doi:10.1016/j.toxrep.2020.08.014)
Supplement: Supplementary file 1 [file mmc1.docx]

**Supplementary Information**

**Predicting PBT and CMR properties of substances of very high concern (SVHCs) using QSAR models and its application for K-REACH**

Joonsik Moon^a^, Byongcheun Lee^b^, Jin-Sung Ra^c,1^, Ki-Tae Kim^a ,^*

Joonsik Moon^a^, Byongcheun Lee^b^, Jinsung Ra^c,1^, Ki-Tae Kim^a ,^*

^a^ Department of Environmental Energy Engineering, Seoul National University of Science and Technology, Seoul 01811, Republic of Korea

^b^ Risk Assessment Division, National Institute of Environmental Research, Incheon 22689, Republic of Korea

^b^ Eco-testing and Risk Assessment Center, Korea Institute of Industrial Technology (KITECH), Ansan 15588, Republic of Korea

^1^Co-corresponding Author

*Corresponding Author:

Ki-Tae Kim, Ph. D.

Department of Environmental Engineering

Seoul National University of Science and Technology

Tel: +82-2-970-6642

Fax: +82-2-970-5776

E-mail: ktkim@seoultech.ac.kr

## **Table S1.** Candidate List of SVHC (download data: 2018-11-11)

| Chemical name | CAS No. | Intrinsic property(ies) referred to in Article 57 | SMILE | Type |
| --- | --- | --- | --- | --- |
| Benzene-1,2,4-tricarboxylic acid 1,2 anhydride | 552-30-7 | Respiratory sensitising properties (Article 57(f) - human health) | O=C(OC(=O)c1ccc(C(=O)O)c2)c12 | Organic |
| Benzo[ghi]perylene | 191-24-2 | PBT (Article 57d)#vPvB (Article 57e) | c16cccc2ccc3ccc4ccc5cccc6c5c4c3c12 | Organic |
| Decamethylcyclopentasiloxane | 541-02-6 | PBT (Article 57d)#vPvB (Article 57e) | C[Si]1(C)O[Si](C)(C)O[Si](C)(C)O[Si](C)(C)O[Si](C)(C)O1 | Organic |
| Dicyclohexyl phthalate | 84-61-7 | Toxic for reproduction (Article 57c)#Endocrine disrupting properties (Article 57(f) - human health) | O=C(OC(CCCC1)C1)c(c(ccc2)C(=O)OC(CCCC3)C3)c2 | Organic |
| Disodium octaborate | 12008-41-2 | Toxic for reproduction (Article 57c) | B(=O)OB1OB2OB(OB(OB(O2)OB(O1)OB=O)[O-])[O-].[Na+].[Na+] | Inorganic |
| Dodecamethylcyclohexasiloxane | 540-97-6 | PBT (Article 57d)#vPvB (Article 57e) | C[Si]1(O[Si](O[Si](O[Si](O[Si](C)(C)O[Si](O1)(C)C)(C)C)(C)C)(C)C)C | Organic |
| Ethylenediamine | 107-15-3 | Respiratory sensitising properties (Article 57(f) - human health) | NCCN | Organic |
| Lead | 7439-92-1 | Toxic for reproduction (Article 57c) | [Pb] | Inorganic |
| Octamethylcyclotetrasiloxane | 556-67-2 | PBT (Article 57d)#vPvB (Article 57e) | C[Si]1(C)O[Si](C)(C)O[Si](C)(C)O[Si](C)(C)O1 | Organic |
| Terphenyl, hydrogenated | 61788-32-7 | vPvB (Article 57e) | C1(C2CCCCC2)CCCC(C3CCCCC3)C1 | Organic |
| 1,6,7,8,9,14,15,16,17,17,18,18-Dodecachloropentacyclo[12.2.1.16,9.02,13.05,10]octadeca-7,15-diene (“Dechlorane Plus”™) | 13560-89-9 | vPvB (Article 57e) | ClC1=C(Cl)C2(Cl)C3CCC4C(CCC3C1(Cl)C2(Cl)Cl)C1(Cl)C(Cl)=C(Cl)C4(Cl)C1(Cl)Cl | Organic |
| Benz[a]anthracene | 56-55-3 | Carcinogenic (Article 57a)#PBT (Article 57d)#vPvB (Article 57e) | C1=CC=C2C(=C1)C=CC3=CC4=CC=CC=C4C=C32 | Organic |
| Cadmium carbonate | 513-78-0 | Carcinogenic (Article 57a)#Mutagenic (Article 57b)#Specific target organ toxicity after repeated exposure (Article 57(f) - human health) | C(=O)([O-])[O-].[Cd+2] | Inorganic |
| Cadmium hydroxide | 21041-95-2 | Carcinogenic (Article 57a)#Mutagenic (Article 57b)#Specific target organ toxicity after repeated exposure (Article 57(f) - human health) | [OH-].[OH-].[Cd++] | Inorganic |
| Cadmium nitrate | 10022-68-1 | Carcinogenic (Article 57a)#Mutagenic (Article 57b)#Specific target organ toxicity after repeated exposure (Article 57(f) - human health) | [N+](=O)([O-])[O-].[N+](=O)([O-])[O-].O.O.O.O.[Cd+2] | Inorganic |
| Chrysene | 218-01-9 | Carcinogenic (Article 57a)#PBT (Article 57d)#vPvB (Article 57e) | c1ccc2ccc3c4ccccc4ccc3c2c1 | Organic |
| Perfluorohexane-1-sulphonic acid | 375-95-1 | vPvB (Article 57e) | O=C(O)C(F)(F)C(F)(F)C(F)(F)C(F)(F)C(F)(F)C(F)(F)C(F)(F)C(F)(F)F | Organic |
| 4,4'-isopropylidenediphenol | 80-05-7 | Toxic for reproduction (Article 57c)#Endocrine disrupting properties (Article 57(f) - environment)#Endocrine disrupting properties (Article 57(f) - human health) | Oc(ccc(c1)C(c(ccc(O)c2)c2)(C)C)c1 | Organic |
| 4-heptylphenol | 1987-50-4 | Endocrine disrupting properties (Article 57(f) - environment) | CCCCCCCC1=CC=C(C=C1)O | Organic |
| Nonadecafluorodecanoic acid (PFDA) | 335-76-2 | Toxic for reproduction (Article 57c)#PBT (Article 57d) | O=C(O)C(F)(F)C(F)(F)C(F)(F)C(F)(F)C(F)(F)C(F)(F)C(F)(F)C(F)(F)C(F)(F)F | Organic |
| p-(1,1-dimethylpropyl)phenol | 80-46-6 | Endocrine disrupting properties (Article 57(f) - environment) | Oc(ccc(c1)C(CC)(C)C)c1 | Organic |
| Benzo[def]chrysene (Benzo[a]pyrene) | 50-32-8 | Carcinogenic (Article 57a)#Mutagenic (Article 57b)#Toxic for reproduction (Article 57c)#PBT (Article 57d)#vPvB (Article 57e) | c(c(c(cc1)ccc2)c2cc3)(c3cc(c4ccc5)c5)c14 | Organic |
| 1,3-propanesultone | 1120-71-4 | Carcinogenic (Article 57a) | O=S(=O)(OCC1)C1 | Organic |
| 2,4-di-tert-butyl-6-(5-chlorobenzotriazol-2-yl)phenol (UV-327) | 3864-99-1 | vPvB (Article 57e) | Oc(c(cc(c1)C(C)(C)C)C(C)(C)C)c1n(nc(c2cc(c3)Cl)c3)n2 | Organic |
| 2-(2H-benzotriazol-2-yl)-4-(tert-butyl)-6-(sec-butyl)phenol (UV-350) | 36437-37-3 | vPvB (Article 57e) | n1c2ccccc2nn1c3cc(C(C)(C)C)cc(C(CC)C)c3O | Organic |
| Nitrobenzene | 98-95-3 | Toxic for reproduction (Article 57c) | N(=O)(=O)c(cccc1)c1 | Organic |
| Perfluorononan-1-oic-acid | 375-95-1- | Toxic for reproduction (Article 57c)#PBT (Article 57d) | O=C(O)C(F)(F)C(F)(F)C(F)(F)C(F)(F)C(F)(F)C(F)(F)C(F)(F)C(F)(F)F | Organic |
| 1,2-benzenedicarboxylic acid, di-C6-10-alkyl esters | 68515-51-5 | Toxic for reproduction (Article 57c) | CCOC(=O)C1=C(C=CC=C1)C(=O)OCC | Organic |
| 2-(2H-benzotriazol-2-yl)-4,6-ditertpentylphenol (UV-328) | 25973-55-1 | PBT (Article 57d)#vPvB (Article 57e) | Oc(c(cc(c1)C(CC)(C)C)C(CC)(C)C)c1n(nc(c2ccc3)c3)n2 | Organic |
| 2-benzotriazol-2-yl-4,6-di-tert-butylphenol (UV-320) | 3846-71-7 | PBT (Article 57d)#vPvB (Article 57e) | Oc(c(cc(c1)C(C)(C)C)C(C)(C)C)c1n(nc(c2ccc3)c3)n2 | Organic |
| 2-ethylhexyl 10-ethyl-4,4-dioctyl-7-oxo-8-oxa-3,5-dithia-4-stannatetradecanoate (DOTE) | 15571-58-1 | Toxic for reproduction (Article 57c) | O=C(CS[Sn](SCC(=O)OCC(CC)CCCC)(CCCCCCCC)CCCCCCCC)OCC(CC)CCCC | Organic |
| Cadmium fluoride | 7790-79-6 | Carcinogenic (Article 57a)#Mutagenic (Article 57b)#Toxic for reproduction (Article 57c)#Specific target organ toxicity after repeated exposure (Article 57(f) - human health) | F[Cd]F | Inorganic |
| Cadmium sulphate | 10124-36-4 | Carcinogenic (Article 57a)#Mutagenic (Article 57b)#Toxic for reproduction (Article 57c)#Specific target organ toxicity after repeated exposure (Article 57(f) - human health) | [Cd++].[O-]S([O-])(=O)=O | Inorganic |
| 1,2-Benzenedicarboxylic acid | 68515-50-4 | Toxic for reproduction (Article 57c) | O=C(c1ccccc1C(=O)OCCCCCC)OCCC(C)(C)C | Organic |
| Cadmium chloride | 10108-64-2 | Carcinogenic (Article 57a)#Mutagenic (Article 57b)#Toxic for reproduction (Article 57c)#Specific target organ toxicity after repeated exposure (Article 57(f) - human health) | Cl[Cd]Cl | Inorganic |
| Sodium perborate | 15120-21-5 | Toxic for reproduction (Article 57c) | [B-]1(OO[B-](OO1)(O)O)(O)O.[Na+].[Na+] | Inorganic |
| Sodium peroxometaborate | 7632-04-4 | Toxic for reproduction (Article 57c) | O=B(=O)[O-].[Na+] | Inorganic |
| Cadmium sulphide | 1306-23-6 | Carcinogenic (Article 57a)#Specific target organ toxicity after repeated exposure (Article 57(f) - human health) | [Cd]=S | Inorganic |
| Dihexyl phthalate | 84-75-3 | Toxic for reproduction (Article 57c) | O=C(OCCCCCC)c(c(ccc1)C(=O)OCCCCCC)c1 | Organic |
| Disodium 3,3'-[[1,1'-biphenyl]-4,4'-diylbis(azo)]bis(4-aminonaphthalene-1-sulphonate) (C.I. Direct Red 28) | 573-58-0 | Carcinogenic (Article 57a) | C1=CC=C2C(=C1)C(=CC(=C2N)N=NC3=CC=C(C=C3)C4=CC=C(C=C4)N=NC5=C(C6=CC=CC=C6C(=C5)S(=O)(=O)[O-])N)S(=O)(=O)[O-].[Na+].[Na+] | Organic |
| Disodium 4-amino-3-[[4'-[(2,4-diaminophenyl)azo][1,1'-biphenyl]-4-yl]azo] -5-hydroxy-6-(phenylazo)naphthalene-2,7-disulphonate (C.I. Direct Black 38) | 1937-37-7 | Carcinogenic (Article 57a) | C1=CC=C(C=C1)NN=C2C(=CC3=CC(=C(C(=C3C2=O)N)N=NC4=CC=C(C=C4)C5=CC=C(C=C5)N=NC6=C(C=C(C=C6)N)N)S(=O)(=O)[O-])S(=O)(=O)[O-].[Na+].[Na+] | Organic |
| Imidazolidine-2-thione (2-imidazoline-2-thiol) | 96-45-7 | Toxic for reproduction (Article 57c) | N(C(=S)NC1)C1 | Organic |
| Lead di(acetate) | 301-04-2 | Toxic for reproduction (Article 57c) | [PbH2++].CC([O-])=O.CC([O-])=O | Inorganic |
| Trixylyl phosphate | 25155-23-1 | Toxic for reproduction (Article 57c) | O=P(Oc1(c(C)cc(C)cc1))(Oc2(cc(C)cc(C)c2))Oc3(c(C)c(C)ccc3) | Organic |
| 4-Nonylphenol, ethoxylated | 26027-38-3 | Endocrine disrupting properties (Article 57(f) - environment) | c1(ccc(OCCO)cc1)CCCCCCCCC | Organic |
| Ammonium pentadecafluorooctanoate (APFO) | 3825-26-1 | Toxic for reproduction (Article 57c)#PBT (Article 57d) | N(H)(H)(H)(H)OC(=O)C(F)(F)C(F)(F)C(F)(F)C(F)(F)C(F)(F)C(F)(F)C(F)(F)F | Organic |
| Cadmium | 7440-43-9 | Carcinogenic (Article 57a)#Specific target organ toxicity after repeated exposure (Article 57(f) - human health) | [Cd] | Inorganic |
| Cadmium oxide | 1306-19-0 | Carcinogenic (Article 57a)#Specific target organ toxicity after repeated exposure (Article 57(f) - human health) | O=[Cd] | Inorganic |
| Dipentyl phthalate (DPP) | 131-18-0 | Toxic for reproduction (Article 57c) | O=C(OCCCCC)c(c(ccc1)C(=O)OCCCCC)c1 | Organic |
| Pentadecafluorooctanoic acid (PFOA) | 335-67-1 | Toxic for reproduction (Article 57c)#PBT (Article 57d) | O=C(O)C(F)(F)C(F)(F)C(F)(F)C(F)(F)C(F)(F)C(F)(F)C(F)(F)F | Organic |
| 1,2-Benzenedicarboxylic acid | 84777-06-0 | Toxic for reproduction (Article 57c) | C1=CC=C(C(=C1)C(=O)O)C(=O)O | Organic |
| 1,2-diethoxyethane | 629-14-1 | Toxic for reproduction (Article 57c) | O(CCOCC)CC | Organic |
| 1-bromopropane (n-propyl bromide) | 106-94-5 | Toxic for reproduction (Article 57c) | BrCCC | Organic |
| 3-ethyl-2-methyl-2-(3-methylbutyl)-1,3-oxazolidine | 143860-04-2 | Toxic for reproduction (Article 57c) | CCN1CCOC1(C)CCC(C)C | Organic |
| 4,4'-methylenedi-o-toluidine | 838-88-0 | Carcinogenic (Article 57a) | Cc2cc(Cc1ccc(N)c(C)c1)ccc2N | Organic |
| 4,4'-oxydianiline | 101-80-4 | Carcinogenic (Article 57a)#Mutagenic (Article 57b) | O(c(ccc(N)c1)c1)c(ccc(N)c2)c2 | Organic |
| 4-(1,1,3,3-tetramethylbutyl)phenol | 140-66-9 | Endocrine disrupting properties (Article 57(f) - environment) | Oc(ccc(c1)C(CC(C)(C)C)(C)C)c1 | Organic |
| 4-aminoazobenzene | 60-09-3 | Carcinogenic (Article 57a) | N(=Nc(cccc1)c1)c(ccc(N)c2)c2 | Organic |
| 4-methyl-m-phenylenediamine (toluene-2,4-diamine) | 95-80-7 | Carcinogenic (Article 57a) | Nc(c(ccc1N)C)c1 | Organic |
| 4-Nonylphenol | 25154-52-3 | Endocrine disrupting properties (Article 57(f) - environment) | CCCCCCCCCC1=CC=C(C=C1)O | Organic |
| 6-methoxy-m-toluidine (p-cresidine) | 120-71-8 | Carcinogenic (Article 57a) | O(c(c(N)cc(c1)C)c1)C | Organic |
| [Phthalato(2-)]dioxotrilead | 69011-06-9 | Toxic for reproduction (Article 57c) | C1=CC=C(C(=C1)C(=O)[O-])C(=O)[O-].[O-2].[O-2].[Pb+2].[Pb+2].[Pb+2] | Other |
| Acetic acid, lead salt, basic | 51404-69-4 | Toxic for reproduction (Article 57c) | CC(=O)O[Pb]OC(=O)C.O.O.[Pb] | Inorganic |
| Biphenyl-4-ylamine | 92-67-1 | Carcinogenic (Article 57a) | Nc(ccc(c(cccc1)c1)c2)c2 | Organic |
| Bis(pentabromophenyl) ether (decabromodiphenyl ether) (DecaBDE) | 1163-19-5 | PBT (Article 57d)#vPvB (Article 57e) | O(c(c(c(c(c1Br)Br)Br)Br)c1Br)c(c(c(c(c2Br)Br)Br)Br)c2Br | Organic |
| trans-cyclohexane-1,2-dicarboxylic anhydride | 14166-21-3 | Respiratory sensitising properties (Article 57(f) - human health) | C1CCC2C(C1)C(=O)OC2=O | Organic |
| Diazene-1,2-dicarboxamide (C,C'-azodi(formamide)) (ADCA) | 123-77-3 | Respiratory sensitising properties (Article 57(f) - human health) | O=C(N=NC(=O)N)N | Organic |
| Dibutyltin dichloride (DBTC) | 683-18-1 | Toxic for reproduction (Article 57c) | Cl[Sn](Cl)(CCCC)CCCC | Inorganic |
| Diethyl sulphate | 64-67-5 | Carcinogenic (Article 57a)#Mutagenic (Article 57b) | O=S(=O)(OCC)OCC | Organic |
| Diisopentyl phthalate | 605-50-5 | Toxic for reproduction (Article 57c) | CC(C)CCOC(=O)c1c(C(=O)OCCC(C)C)cccc1 | Organic |
| Dimethyl sulphate | 77-78-1 | Carcinogenic (Article 57a) | O=S(=O)(OC)OC | Organic |
| Dinoseb (6-sec-butyl-2,4-dinitrophenol) | 88-85-7 | Toxic for reproduction (Article 57c) | N(=O)(=O)c(cc(N(=O)(=O))c(O)c1C(CC)C)c1 | Organic |
| Dioxobis(stearato)trilead | 12578-12-0 | Toxic for reproduction (Article 57c) | CCCCCCCCCCCCCCCCCC(=O)O[Pb]O[Pb]O[Pb]OC(=O)CCCCCCCCCCCCCCCCC | Organometallic |
| Furan | 110-00-9 | Carcinogenic (Article 57a) | O1C=CC=C1 | Organic |
| Henicosafluoroundecanoic acid | 2058-94-8 | vPvB (Article 57e) | OC(=O)C(F)(F)C(F)(F)C(F)(F)C(F)(F)C(F)(F)C(F)(F)C(F)(F)C(F)(F)C(F)(F)C(F)(F)F | Organic |
| Heptacosafluorotetradecanoic acid | 376-06-7 | vPvB (Article 57e) | O=C(O)C(F)(F)C(F)(F)C(F)(F)C(F)(F)C(F)(F)C(F)(F)C(F)(F)C(F)(F)C(F)(F)C(F)(F)C(F)(F)C(F)(F)C(F)(F)F | Organic |
| Hexahydro-1-methylphthalic anhydride | 48122-14-1 | Respiratory sensitising properties (Article 57(f) - human health) | CC12CCCCC1C(=O)OC2=O | Organic |
| Lead bis(tetrafluoroborate) | 13814-96-5 | Toxic for reproduction (Article 57c) | [B-](F)(F)(F)F.[B-](F)(F)(F)F.[Pb+2] | Inorganic |
| Lead cyanamidate | 20837-86-9 | Toxic for reproduction (Article 57c) | C(#N)N.[Pb] | Inorganic |
| Lead dinitrate | 10099-74-8 | Toxic for reproduction (Article 57c) | [Pb](ON(=O)=O)ON(=O)=O | Inorganic |
| Lead monoxide (lead oxide) | 1317-36-8 | Toxic for reproduction (Article 57c) | [Pb]=O | Inorganic |
| Lead oxide sulfate | 12036-76-9 | Toxic for reproduction (Article 57c) | [O-]S(=O)(=O)[O-].O=[Pb].[Pb+2] | Inorganic |
| Lead titanium trioxide | 12060-00-3 | Toxic for reproduction (Article 57c) | [O-][Ti](=O)[O-].[Pb+2] | Inorganic |
| Lead titanium zirconium oxide | 12626-81-2 | Toxic for reproduction (Article 57c) | [O-2].[O-2].[O-2].[O-2].[O-2].[Ti+4].[Zr+4].[Pb+2] | Inorganic |
| Methoxyacetic acid | 625-45-6 | Toxic for reproduction (Article 57c) | O=C(O)COC | Organic |
| Methyloxirane (Propylene oxide) | 75-56-9 | Carcinogenic (Article 57a)#Mutagenic (Article 57b) | O(C1C)C1 | Organic |
| N,N-dimethylformamide | 68-12-2 | Toxic for reproduction (Article 57c) | O=CN(C)C | Organic |
| N-methylacetamide | 79-16-3 | Toxic for reproduction (Article 57c) | O=C(NC)C | Organic |
| N-pentyl-isopentylphthalate | 776297-69-9 | Toxic for reproduction (Article 57c) | CCCCCOC(=O)C1=CC=CC=C1C(=O)OCCC(C)C | Organic |
| o-aminoazotoluene | 97-56-3 | Carcinogenic (Article 57a) | N(=Nc(c(ccc1)C)c1)c(ccc(N)c2C)c2 | Organic |
| o-toluidine | 95-53-4 | Carcinogenic (Article 57a) | Nc(c(ccc1)C)c1 | Organic |
| Orange lead (lead tetroxide) | 1314-41-6 | Toxic for reproduction (Article 57c) | O1[Pb]O[Pb]12O[Pb]O2 | Inorganic |
| Pentacosafluorotridecanoic acid | 72629-94-8 | vPvB (Article 57e) | O=C(O)C(F)(F)C(F)(F)C(F)(F)C(F)(F)C(F)(F)C(F)(F)C(F)(F)C(F)(F)C(F)(F)C(F)(F)C(F)(F)C(F)(F)F | Organic |
| Pentalead tetraoxide sulphate | 12065-90-6 | Toxic for reproduction (Article 57c) | O.O.O.O.[O-]S(=O)(=O)[O-].[Pb].[Pb].[Pb].[Pb].[Pb] | Inorganic |
| Pyrochlore, antimony lead yellow | 8012-00-8 | Toxic for reproduction (Article 57c) | [Pb++].[Pb++].[O-][Sb]([O-])(=O)O[Sb]([O-])([O-])=O | Inorganic |
| Silicic acid (H2Si2O5), barium salt (1:1), lead-doped | 68784-75-8 | Toxic for reproduction (Article 57c) | [Ba++].[PbH2].[O-][Si](=O)O[Si]([O-])=O | Inorganic |
| Silicic acid, lead salt | 11120-22-2 | Toxic for reproduction (Article 57c) | [Pb++].[O-][Si]([O-])=O | Inorganic |
| Sulfurous acid, lead salt, dibasic | 62229-08-7 | Toxic for reproduction (Article 57c) | [OH-].[OH-].[Pb++].[Pb++].[O-]S([O-])=O | Inorganic |
| Tetraethyllead | 78-00-2 | Toxic for reproduction (Article 57c) | CC[Pb](CC)(CC)CC | Organicanometallic |
| Tetralead trioxide sulphate | 12202-17-4 | Toxic for reproduction (Article 57c) | [O--].[O--].[O--].[Pb++].[Pb++].[Pb++].[Pb++].[O-]S([O-])(=O)=O | Inorganic |
| Tricosafluorododecanoic acid | 307-55-1 | vPvB (Article 57e) | O=C(O)C(F)(F)C(F)(F)C(F)(F)C(F)(F)C(F)(F)C(F)(F)C(F)(F)C(F)(F)C(F)(F)C(F)(F)C(F)(F)F | Organic |
| Trilead bis(carbonate) dihydroxide | 1319-46-6 | Toxic for reproduction (Article 57c) | [OH-].[OH-].[Pb++].[Pb++].[Pb++].[O-]C([O-])=O.[O-]C([O-])=O | Inorganic |
| Trilead dioxide phosphonate | 12141-20-7 | Toxic for reproduction (Article 57c) | [O--].[O--].[Pb++].[Pb++].[Pb++].[O-]P([O-])=O | Inorganic |
| 1,2-bis(2-methoxyethoxy)ethane (TEGDME,triglyme) | 112-49-2 | Toxic for reproduction (Article 57c) | O(CCOCCOC)CCOC | Organic |
| 1,2-dimethoxyethane,ethylene glycol dimethyl ether (EGDME) | 110-71-4 | Toxic for reproduction (Article 57c) | O(CCOC)C | Organic |
| 1,3,5-Tris(oxiran-2-ylmethyl)-1,3,5-triazinane-2,4,6-trione (TGIC) | 2451-62-9 | Mutagenic (Article 57b) | O=C(N(C(=O)N(C1=O)CC(O2)C2)CC(O3)C3)N1CC(O4)C4 | Organic |
| 1,3,5-tris[(2S and 2R)-2,3-epoxypropyl]-1,3,5-triazine-2,4,6-(1H,3H,5H)-trione (β-TGIC) | 59653-74-6 | Mutagenic (Article 57b) | C1C(O1)CN2C(=O)N(C(=O)N(C2=O)CC3CO3)CC4CO4 | Organic |
| 4,4'-bis(dimethylamino)-4''-(methylamino)trityl alcohol | 561-41-1 | Carcinogenic (Article 57a) | CN(C)c1ccc(C(O)(c2ccc(NC)cc2)c2ccc(N(C)C)cc2)cc1 | Organic |
| 4,4'-bis(dimethylamino)benzophenone (Michler’s ketone) | 90-94-8 | Carcinogenic (Article 57a) | O=C(c(ccc(N(C)C)c1)c1)c(ccc(N(C)C)c2)c2 | Organic |
| [4-[4,4'-bis(dimethylamino) benzhydrylidene]cyclohexa-2,5-dien-1-ylidene]dimethylammonium chloride  (C.I. Basic Violet 3) | 548-62-9 | Carcinogenic (Article 57a) | CN(C)c1ccc(cc1)C(=C2C=CC(C=C2)=[N+](C)C)c3ccc(cc3)N(C)C.[Cl-] | Organic |
| [4-[[4-anilino-1-naphthyl][4-(dimethylamino)phenyl]methylene]cyclohexa-2,5-dien-1-ylidene] dimethylammonium chloride (C.I. Basic Blue 26) | 2580-56-5 | Carcinogenic (Article 57a) | c(ccc1Nc(ccc2C(=C(C=CC3=N(Cl)(C)C)C=C3)c(ccc4N(C)C)cc4)c(ccc5)c2c5)cc1 | Organic |
| Diboron trioxide | 1303-86-2 | Toxic for reproduction (Article 57c) | OB(O)O | Inorganic |
| Formamide | 75-12-7 | Toxic for reproduction (Article 57c) | O=CN | Organic |
| Lead(II) bis(methanesulfonate) | 17570-76-2 | Toxic for reproduction (Article 57c) | [PbH2++].CS([O-])(=O)=O.CS([O-])(=O)=O | Inorganic |
| N,N,N',N'-tetramethyl-4,4'-methylenedianiline (Michler’s base) | 101-61-1 | Carcinogenic (Article 57a) | N(c(ccc(c1)Cc(ccc(N(C)C)c2)c2)c1)(C)C | Organic |
| α,α-Bis[4-(dimethylamino)phenyl]-4 (phenylamino)naphthalene-1-methanol (C.I. Solvent Blue 4) | 6786-83-0 | Carcinogenic (Article 57a) | N(c(ccc(c1)C(O)(c(ccc(N(C)C)c2)c2)c(c(c(c(Nc(cccc3)c3)c4)ccc5)c5)c4)c1)(C)C | Organic |
| 1,2-dichloroethane | 107-06-2 | Carcinogenic (Article 57a) | ClCCCl | Organic |
| 2,2'-dichloro-4,4'-methylenedianiline | 101-14-4 | Carcinogenic (Article 57a) | Nc(c(cc(c1)Cc(ccc(N)c2Cl)c2)Cl)c1 | Organic |
| 2-Methoxyaniline, o-Anisidine | 90-04-0 | Carcinogenic (Article 57a) | O(c(c(N)ccc1)c1)C | Organic |
| 4-(1,1,3,3-tetramethylbutyl)phenol | 140-66-9 | Endocrine disrupting properties (Article 57(f) - environment) | Oc(ccc(c1)C(CC(C)(C)C)(C)C)c1 | Organic |
| Arsenic acid | 7778-39-4 | Carcinogenic (Article 57a) | O=[As](O)(O)O | Inorganic |
| Bis(2-methoxyethyl) ether | 111-96-6 | Toxic for reproduction (Article 57c) | O(CCOC)CCOC | Organic |
| Bis(2-methoxyethyl) phthalate | 117-82-8 | Toxic for reproduction (Article 57c) | O=C(OCCOC)c(c(ccc1)C(=O)OCCOC)c1 | Organic |
| Calcium arsenate | 7778-44-1 | Carcinogenic (Article 57a) | [Ca++].[Ca++].[Ca++].[O-][As]([O-])([O-])=O.[O-][As]([O-])([O-])=O | Inorganic |
| Dichromium tris(chromate) | 24613-89-6 | Carcinogenic (Article 57a) | [Cr+3].[Cr+3].[O-][Cr]([O-])(=O)=O.[O-][Cr]([O-])(=O)=O.[O-][Cr]([O-])(=O)=O | Inorganic |
| Formaldehyde, oligomeric reaction products with aniline | 25214-70-4 | Carcinogenic (Article 57a) | O=Cc1cc(ccc1)N | Organic |
| Lead diazide, Lead azide | 13424-46-9 | Toxic for reproduction (Article 57c) | [PbH2++].[N-]=[N+]=[N-].[N-]=[N+]=[N-] | Inorganic |
| Lead dipicrate | 6477-64-1 | Toxic for reproduction (Article 57c) | C1=C(C=C(C(=C1[N+](=O)[O-])[O-])[N+](=O)[O-])[N+](=O)[O-].C1=C(C=C(C(=C1[N+](=O)[O-])[O-])[N+](=O)[O-])[N+](=O)[O-].[Pb+2] | Organometallic |
| Lead styphnate | 15245-44-0 | Toxic for reproduction (Article 57c) | [O-][N+](=O)c1cc(c2O[Pb]Oc1c2[N+]([O-])=O)[N+]([O-])=O | Organometallic |
| N,N-dimethylacetamide | 127-19-5 | Toxic for reproduction (Article 57c) | O=C(N(C)C)C | Organic |
| Pentazinc chromate octahydroxide | 49663-84-5 | Carcinogenic (Article 57a) | [OH-].[OH-].[Zn++].[Zn++].[Zn++].[O-][Cr]([O-])(=O)=O.[O-][Cr]([O-])(=O)=O | Inorganic |
| Phenolphthalein | 77-09-8 | Carcinogenic (Article 57a) | O=C(OC(c1cccc2)(c(ccc(O)c3)c3)c(ccc(O)c4)c4)c12 | Organic |
| Potassium hydroxyoctaoxodizincatedichromate | 11103-86-9 | Carcinogenic (Article 57a) | [OH-].[K+].[Zn++].[Zn++].[O-][Cr]([O-])(=O)=O.[O-][Cr]([O-])(=O)=O | Inorganic |
| Trilead diarsenate | 3687-31-8 | Carcinogenic (Article 57a)#Toxic for reproduction (Article 57c) | [O-][As](=O)([O-])[O-].[O-][As](=O)([O-])[O-].[Pb+2].[Pb+2].[Pb+2] | Inorganic |
| 1,2,3-trichloropropane | 96-18-4 | Carcinogenic (Article 57a)#Toxic for reproduction (Article 57c) | ClCC(Cl)CCl | Organic |
| 1,2-Benzenedicarboxylic acid, di-C6-8-branched alkyl esters, C7-rich | 71888-89-6 | Toxic for reproduction (Article 57c) | O=C(c1ccccc1C(=O)OCCCCC(C)C)OCCCCC(C)C | Organic |
| 1,2-Benzenedicarboxylic acid, di-C7-11-branched and linear alkyl esters | 68515-42-4 | Toxic for reproduction (Article 57c) | O=C(c1ccccc1C(=O)OCCCCCC(C)C)OCCCCCCCC | Organic |
| 1-Methyl-2-pyrrolidone (NMP) | 872-50-4 | Toxic for reproduction (Article 57c) | O=C1CCCN1C | Organic |
| 2-ethoxyethyl acetate | 111-15-9 | Toxic for reproduction (Article 57c) | O=C(OCCOCC)C | Organic |
| Hydrazine | 302-01-2 | Carcinogenic (Article 57a) | NN | Inorganic |
| Strontium chromate | 7789-06-2 | Carcinogenic (Article 57a) | O=[Cr]1(=O)O[Sr]O1 | Inorganic |
| 2-ethoxyethanol | 110-80-5 | Toxic for reproduction (Article 57c) | O(CCO)CC | Organic |
| 2-methoxyethanol | 109-86-4 | Toxic for reproduction (Article 57c) | O(CCO)C | Organic |
| Dichromic acid | 7738-94-5 | Carcinogenic (Article 57a) | O=[Cr](=O)(O)O | Inorganic |
| Chromium trioxide | 1333-82-0 | Carcinogenic (Article 57a)#Mutagenic (Article 57b) | [Cr](=O)(=O)=O | Inorganic |
| Cobalt(II) carbonate | 513-79-1 | Carcinogenic (Article 57a)#Toxic for reproduction (Article 57c) | C(=O)([O-])[O-].[Co+2] | Inorganic |
| Cobalt(II) diacetate | 71-48-7 | Carcinogenic (Article 57a)#Toxic for reproduction (Article 57c) | CC(=O)[O-].CC(=O)[O-].[Co+2] | Inorganic |
| Cobalt(II) dinitrate | 10141-05-6 | Carcinogenic (Article 57a)#Toxic for reproduction (Article 57c) | O=N(=O)O[Co] | Inorganic |
| Cobalt(II) sulphate | 10124-43-3 | Carcinogenic (Article 57a)#Toxic for reproduction (Article 57c) | O1S(=O)(=O)O[Co]1 | Inorganic |
| Ammonium dichromate | 7789-09-5 | Carcinogenic (Article 57a)#Mutagenic (Article 57b)#Toxic for reproduction (Article 57c) | [NH4+].[NH4+].[O-][Cr](=O)(=O)O[Cr]([O-])(=O)=O | Inorganic |
| Boric acid | 10043-35-3 | Toxic for reproduction (Article 57c) | OB(O)O | Inorganic |
| Disodium tetraborate, anhydrous | 1303-96-4 | Toxic for reproduction (Article 57c) | B1(OB2OB(OB(O1)O2)[O-])[O-].O.O.O.O.O.O.O.O.O.O.[Na+].[Na+] | Inorganic |
| Potassium chromate | 7789-00-6 | Carcinogenic (Article 57a)#Mutagenic (Article 57b) | [K]O[Cr](=O)(=O)O[K] | Inorganic |
| Potassium dichromate | 7778-50-9 | Carcinogenic (Article 57a)#Mutagenic (Article 57b)#Toxic for reproduction (Article 57c) | [K]O[Cr](=O)(=O)O[Cr](=O)(=O)O[K] | Inorganic |
| Sodium chromate | 7775-11-3 | Carcinogenic (Article 57a)#Mutagenic (Article 57b)#Toxic for reproduction (Article 57c) | [Na+].[Na+].[O-][Cr]([O-])(=O)=O | Inorganic |
| Tetraboron disodium heptaoxide, hydrate | 12267-73-1 | Toxic for reproduction (Article 57c) | B1(OB2OB(OB(O1)O2)[O-])[O-].[Na+].[Na+] | Inorganic |
| Trichloroethylene | 79-01-6 | Carcinogenic (Article 57a) | C(=CCl)(Cl)Cl | Organic |
| Acrylamide | 79-06-1 | Carcinogenic (Article 57a)#Mutagenic (Article 57b) | O=C(N)C=C | Organic |
| 2,4-dinitrotoluene | 121-14-2 | Carcinogenic (Article 57a) | N(=O)(=O)c(ccc(c1N(=O)(=O))C)c1 | Organic |
| Diisobutyl phthalate | 84-69-5 | Toxic for reproduction (Article 57c)#Endocrine disrupting properties (Article 57(f) - human health) | O=C(OCC(C)C)c(c(ccc1)C(=O)OCC(C)C)c1 | Organic |
| Lead chromate | 7758-97-6 | Carcinogenic (Article 57a)#Toxic for reproduction (Article 57c) | [O-][Cr](=O)(=O)[O-].[Pb+2] | Inorganic |
| Lead chromate molybdate sulphate red (C.I. Pigment Red 104) | 12656-85-8 | Carcinogenic (Article 57a)#Toxic for reproduction (Article 57c) | [Pb++].[Pb++].[Pb++].[O-]S([O-])(=O)=O.[O-][Cr]([O-])(=O)=O.[O-][Mo]([O-])(=O)=O | Inorganic |
| Lead sulfochromate yellow  (C.I. Pigment Yellow 34) | 1344-37-2 | Carcinogenic (Article 57a)#Toxic for reproduction (Article 57c) | PbH2++].[O-][Cr]([O-])(=O)=O | Inorganic |
| Tris(2-chloroethyl) phosphate | 115-96-8 | Toxic for reproduction (Article 57c) | O=P(OCCCl)(OCCCl)OCCCl | Organic |
| 4,4'- Diaminodiphenylmethane (MDA) | 101-77-9 | Carcinogenic (Article 57a) | Nc(ccc(c1)Cc(ccc(N)c2)c2)c1 | Organic |
| 5-tert-butyl-2,4,6-trinitro-m-xylene (Musk xylene) | 81-15-2 | vPvB (Article 57e) | N(=O)(=O)c(c(c(N(=O)(=O))c(c1N(=O)(=O))C(C)(C)C)C)c1C | Organic |
| Anthracene | 120-12-7 | PBT (Article 57d) | c(c(ccc1)cc(c2ccc3)c3)(c1)c2 | Organic |
| Benzyl butyl phthalate (BBP) | 85-68-7 | Toxic for reproduction (Article 57c)#Endocrine disrupting properties (Article 57(f) - human health) | O=C(OCc(cccc1)c1)c(c(ccc2)C(=O)OCCCC)c2 | Organic |
| Bis (2-ethylhexyl)phthalate (DEHP) | 117-81-7 | Toxic for reproduction (Article 57c)#Endocrine disrupting properties (Article 57(f) - environment)#Endocrine disrupting properties (Article 57(f) - human health) | O=C(OCC(CCCC)CC)c(c(ccc1)C(=O)OCC(CCCC)CC)c1 | Organic |
| Bis(tributyltin) oxide (TBTO) | 56-35-9 | PBT (Article 57d) | CCCC[Sn](CCCC)(CCCC)O[Sn](CCCC)(CCCC)CCCC | Organometallic |
| Cobalt dichloride | 7646-79-9 | Carcinogenic (Article 57a)#Toxic for reproduction (Article 57c) | [Co](Cl)Cl | Inorganic |
| Diarsenic pentaoxide | 1303-28-2 | Carcinogenic (Article 57a) | O=[As](=O)O[As](=O)=O | Inorganic |
| Diarsenic trioxide | 1327-53-3 | Carcinogenic (Article 57a) | O=[As]O[As]=O | Inorganic |
| Dibutyl phthalate (DBP) | 84-74-2 | Toxic for reproduction (Article 57c)#Endocrine disrupting properties (Article 57(f) - human health) | O=C(OCCCC)c(c(ccc1)C(=O)OCCCC)c1 | Organic |
| Hexabromocyclododecane | 25637-99-4 | PBT (Article 57d) | BrC1CC(CC(CC(CC(CC(C1)Br)Br)Br)Br)Br | Organic |
| Lead hydrogen arsenate | 7784-40-9 | Carcinogenic (Article 57a)#Toxic for reproduction (Article 57c) | O=[As](O)(O)O[Pb] | Inorganic |
| Sodium dichromate | 10588-01-9 | Carcinogenic (Article 57a)#Mutagenic (Article 57b)#Toxic for reproduction (Article 57c) | O.O.[Na+].[Na+].[O-][Cr](=O)(=O)O[Cr]([O-])(=O)=O | Inorganic |
| Triethyl arsenate | 15606-95-8 | Carcinogenic (Article 57a) | CCO[As](=O)(OCC)OCC | Organic |

**Table S2.** Details on the results of predicted and measured values using various QSAR models, and WoE approach.

| Chemical Name | CAS no. | QSAR models | | | | | | | | | | | | |
| --- | --- | --- | --- | --- | --- | --- | --- | --- | --- | --- | --- | --- | --- | --- |
|  |  | KOWWIN | BIOWIN | BCFBAF | HYDROWIN | WoE | Toxtree | | LAZAR | | CAESAE | | WoE | |
|  |  |  |  |  |  |  | C | M | C | M | C | M | C | M |
| Benzene-1,2,4-tricarboxylic acid 1,2 anhydride | 552-30-7 | TN | TN | TN | TN | TN | TN | TN | TN | TN | TN | TN | TN | TN |
| Benzo[ghi]perylene | 191-24-2 | TP | TP | TP | NA | TP | FP | FP | FP | FP | FP | FP | FP | FP |
| Decamethylcyclopentasiloxane | 541-02-6 | FN | TP | TP | NA | TP | TN | TN | NA | NA | TN | TN | TN | TN |
| Dicyclohexyl phthalate | 84-61-7 | FP | TN | FP | FP | FP | FP | TN | TN | TN | TN | TN | TN | TN |
| Disodium octaborate | 12008-41-2 | NA | NA | NA | NA | NA | TN | TN | NA | NA | NA | NA | TN | TN |
| Dodecamethylcyclohexasiloxane | 540-97-6 | FN | TP | TN | NA | TP | TN | TN | NA | NA | TN | TN | TN | TN |
| Ethylenediamine | 107-15-3 | TN | TN | TN | NA | TN | TN | TN | FP | TN | FP | TN | FP | TN |
| Lead | 7439-92-1 | NA | NA | NA | NA | NA | TN | TN | TN | NA | NA | NA | TN | TN |
| Octamethylcyclotetrasiloxane | 556-67-2 | TP | TP | TP | NA | TP | TN | TN | NA | NA | TN | TN | TN | TN |
| Terphenyl, hydrogenated | 61788-32-7 | TP | TP | TP | NA | TP | TN | TN | TN | TN | TN | TN | TN | TN |
| 1,6,7,8,9,14,15,16,17,17,18,18-Dodecachloropentacyclo[12.2.1.16,9.02,13.05,10]octadeca-7,15-diene (“Dechlorane Plus”™) | 13560-89-9 | FN | TP | FN | NA | FN | FP | TN | FP | TN | FP | TN | FP | TN |
| Benz[a]anthracene | 56-55-3 | TP | TP | TP | NA | TP | TP | FP | TP | FP | TP | FP | TP | TP |
| Cadmium carbonate | 513-78-0 | NA | NA | NA | NA | NA | FN | FN | NA | FN | FN | NA | FN | FN |
| Cadmium hydroxide | 21041-95-2 | NA | NA | NA | NA | NA | FN | FN | NA | NA | TP | NA | TP | FN |
| Cadmium nitrate | 10022-68-1 | NA | NA | NA | NA | NA | FN | FN | FN | NA | NA | NA | FN | FN |
| Chrysene | 218-01-9 | TP | TP | TP | NA | TP | TP | FP | TP | FP | TP | FP | TP | FP |
| Perfluorohexane-1-sulphonic acid | 375-95-1 | TP | TP | FN | NA | TP | FP | TN | NA | TN | FP | TN | FP | TN |
| 4,4'-isopropylidenediphenol | 80-05-7 | TN | FP | TN | NA | TN | TN | TN | TN | TN | TN | TN | TN | TN |
| 4-heptylphenol | 1987-50-4 | FN | TN | TN | NA | TN | TN | TN | TN | TN | TN | TN | TN | TN |
| Nonadecafluorodecanoic acid (PFDA) | 335-76-2 | TP | TP | FN | NA | TP | FP | TN | NA | TN | FP | TN | FP | TN |
| p-(1,1-dimethylpropyl)phenol | 80-46-6 | TN | FP | TN | NA | TN | TN | TN | TN | TN | TN | TN | TN | TN |
| Benzo[def]chrysene (Benzo[a]pyrene) | 50-32-8 | TP | TP | TP | NA | TP | TP | TP | TP | TP | TP | TP | TP | TP |
| 1,3-propanesultone | 1120-71-4 | TN | TN | TN | NA | TN | TP | FP | FN | FP | TP | FP | TP | FP |
| 2,4-di-tert-butyl-6-(5-chlorobenzotriazol-2-yl)phenol (UV-327) | 3864-99-1 | TP | TP | TP | NA | TP | TN | TN | TN | TN | TN | TN | TN | TN |
| 2-(2H-benzotriazol-2-yl)-4-(tert-butyl)-6-(sec-butyl)phenol (UV-350) | 36437-37-3 | TP | TP | TP | NA | TP | TN | TN | TN | TN | FP | TN | TN | TN |
| Nitrobenzene | 98-95-3 | TN | TN | TN | NA | TN | FP | FP | FP | FP | FP | TN | FP | FP |
| Perfluorononan-1-oic-acid | 375-95-1- | TP | TP | FN | NA | TP | FP | TN | NA | TN | FP | TN | FP | TN |
| 1,2-benzenedicarboxylic acid, di-C6-10-alkyl esters | 68515-51-5 | TN | TN | TN | FP | TN | FP | TN | TN | TN | TN | TN | TN | TN |
| 2-(2H-benzotriazol-2-yl)-4,6-ditertpentylphenol (UV-328) | 25973-55-1 | TP | TP | TP | NA | TP | TN | TN | TN | TN | TN | TN | TN | TN |
| 2-benzotriazol-2-yl-4,6-di-tert-butylphenol (UV-320) | 3846-71-7 | TP | TP | TP | NA | TP | TN | TN | TN | TN | FP | TN | TN | TN |
| 2-ethylhexyl 10-ethyl-4,4-dioctyl-7-oxo-8-oxa-3,5-dithia-4-stannatetradecanoate (DOTE) | 15571-58-1 | FP | TN | TN | FP | TN | FP | TN | FP | TN | TN | TN | FP | TN |
| Cadmium fluoride | 7790-79-6 | NA | NA | NA | NA | NA | FN | FN | NA | NA | TP | NA | TP | FN |
| Cadmium sulphate | 10124-36-4 | NA | NA | NA | NA | NA | FN | FN | TP | NA | FN | NA | FN | FN |
| 1,2-Benzenedicarboxylic acid | 68515-50-4 | FP | TN | TN | NA | TN | FP | TN | TN | TN | TN | TN | FP | TN |
| Cadmium chloride | 10108-64-2 | NA | NA | NA | NA | NA | FN | FN | NA | NA | FP | NA | TP | FN |
| Sodium perborate | 15120-21-5 | NA | NA | NA | NA | NA | TN | TN | TN | NA | NA | NA | TN | TN |
| Sodium peroxometaborate | 7632-04-4 | NA | NA | NA | NA | NA | TN | TN | TN | NA | NA | NA | TN | TN |
| Cadmium sulphide | 1306-23-6 | NA | NA | NA | NA | NA | FN | TN | NA | NA | TP | NA | FP | TNTN |
| Dihexyl phthalate | 84-75-3 | FP | TN | TN | FP | TN | FP | TN | TN | TN | TN | TN | TN | FP |
| Disodium 3,3'-[[1,1'-biphenyl]-4,4'-diylbis(azo)]bis(4-aminonaphthalene-1-sulphonate) (C.I. Direct Red 28) | 573-58-0 | TN | FP | TN | NA | TN | TP | FP | TP | FP | NA | NA | TP | FP |
| Disodium 4-amino-3-[[4'-[(2,4-diaminophenyl)azo][1,1'-biphenyl]-4-yl]azo] -5-hydroxy-6-(phenylazo)naphthalene-2,7-disulphonate (C.I. Direct Black 38) | 1937-37-7 | FP | FP | TN | NA | FP | TP | FP | TN | FP | FN | TN | TP | TN |
| Imidazolidine-2-thione (2-imidazoline-2-thiol) | 96-45-7 | TF | TN | TN | NA | TN | TP | FP | TN | TN | FP | FP | FP | FP |
| Lead di(acetate) | 301-04-2 | NA | NA | NA | NA | TN | TN | TN | TN | FP | TN | NA | TN | TN |
| Trixylyl phosphate | 25155-23-1 | TN | FP | TN | FP | TN | TN | TN | TN | FP | TN | TN | TN | TN |
| 4-Nonylphenol, ethoxylated | 26027-38-3 | FP | TN | TN | NA | TN | TN | TN | TN | TN | TN | TN | TN | TN |
| Ammonium pentadecafluorooctanoate (APFO) | 3825-26-1 | FN | TP | FN | FN | FN | FP | TN | NA | NA | NA | NA | FP | TN |
| Cadmium | 7440-43-9 | NA | NA | NA | NA | NA | FN | TN | FN | NA | NA | NA | FN | TN |
| Cadmium oxide | 1306-19-0 | NA | NA | NA | NA | NA | FN | TN | NA | NA | TP | NA | FP | TN |
| Dipentyl phthalate (DPP) | 131-18-0 | FP | TN | TN | FP | TN | FP | TN | TN | TN | TN | TN | TN | TN |
| Pentadecafluorooctanoic acid (PFOA) | 335-67-1 | TP | TP | FN | NA | TP | FP | TN | NA | TN | FP | TN | FP | TN |
| 1,2-Benzenedicarboxylic acid | 84777-06-0 | TN | TN | TN | NA | TN | FP | TN | TN | TN | TM | TM | TN | TN |
| 1,2-diethoxyethane | 629-14-1 | TN | TN | TN | NA | TN | TN | TN | FP | TN | FP | TN | FP | TN |
| 1-bromopropane (n-propyl bromide) | 106-94-5 | TN | TN | TN | FP | TN | FP | FP | FP | TN | TN | FP | FP | TN |
| 3-ethyl-2-methyl-2-(3-methylbutyl)-1,3-oxazolidine | 143860-04-2 | TN | FP | FP | NA | FP | TN | TN | TN | TN | TN | TN | TN | TN |
| 4,4'-methylenedi-o-toluidine | 838-88-0 | TN | FP | TN | NA | TN | TP | FP | TP | FP | TP | FP | TP | FP |
| 4,4'-oxydianiline | 101-80-4 | TN | FP | TN | NA | TN | TP | TP | TP | TP | TP | TP | TP | TP |
| 4-(1,1,3,3-tetramethylbutyl)phenol | 140-66-9 | FP | FP | FP | NA | FP | TN | TN | TN | TN | TN | TN | TN | TN |
| 4-aminoazobenzene | 60-09-3 | TN | FP | TN | NA | TN | TP | FP | TP | FP | TP | FP | TP | FP |
| 4-methyl-m-phenylenediamine (toluene-2,4-diamine) | 95-80-7 | TN | FP | TN | NA | TN | TP | FP | TP | FP | TP | FP | TP | FP |
| 4-Nonylphenol | 25154-52-3 | FP | TN | TN | NA | TN | TN | TN | TN | TN | TN | TN | TN | TN |
| 6-methoxy-m-toluidine (p-cresidine) | 120-71-8 | TN | FP | TN | NA | TN | TP | TN | TP | FP | TP | FP | TP | FP |
| [Phthalato(2-)]dioxotrilead | 69011-06-9 | NA | NA | NA | NA | NA | FP | TN | TN | TN | NA | NA | FP | TN |
| Acetic acid, lead salt, basic | 51404-69-4 | NA | NA | NA | NA | NA | TN | FP | TN | TN | NA | NA | TN | FP |
| Biphenyl-4-ylamine | 92-67-1 | TN | TN | TN | NA | TN | TP | FP | TP | FP | TP | FP | TP | FP |
| Bis(pentabromophenyl) ether (decabromodiphenyl ether) (DecaBDE) | 1163-19-5 | FN | TP | FN | NA | FN | FP | TN | FP | TN | FP | TN | FP | TN |
| trans-cyclohexane-1,2-dicarboxylic anhydride | 14166-21-3 | TN | TN | TN | TN | TN | TN | TN | TN | TN | TN | FP | TN | TN |
| Diazene-1,2-dicarboxamide (C,C'-azodi(formamide)) (ADCA) | 123-77-3 | TN | TN | TN | NA | TN | FP | FP | FP | TN | FP | FP | FP | FP |
| Dibutyltin dichloride (DBTC) | 683-18-1 | NA | NA | NA | NA | TN | TN | FP | FP | TN | TN | TN | TN | TN |
| Diethyl sulphate | 64-67-5 | TN | TN | TN | NA | TN | TP | TP | TP | TP | FN | FP | TP | TP |
| Diisopentyl phthalate | 605-50-5 | FP | TN | FP | FP | FP | FP | TN | TN | TN | TN | TN | TN | TN |
| Dimethyl sulphate | 77-78-1 | TN | TN | TN | NA | TN | TP | FP | FN | FP | TP | FP | TP | FP |
| Dinoseb (6-sec-butyl-2,4-dinitrophenol) | 88-85-7 | TN | FP | TN | NA | TN | FP | FP | TN | TN | TN | FP | TN | FP |
| Dioxobis(stearato)trilead | 12578-12-0 | NA | NA | NA | NA | NA | TN | TN | FP | TN | TN | NA | TN | TN |
| Furan | 110-00-9 | TN | TN | TN | NA | TN | FN | TN | TP | TN | TP | TN | TP | TN |
| Henicosafluoroundecanoic acid | 2058-94-8 | TP | TP | FN | NA | TP | FP | TN | NA | TN | FP | TN | FP | TN |
| Heptacosafluorotetradecanoic acid | 376-06-7 | FN | TP | FN | NA | FN | FP | TN | NA | TN | TN | TN | FP | TN |
| Hexahydro-1-methylphthalic anhydride | 48122-14-1 | TN | FP | TN | TN | TN | TN | TN | FP | TN | TN | TN | TN | TN |
| Lead bis(tetrafluoroborate) | 13814-96-5 | NA | NA | NA | NA | NA | TN | TN | TN | NA | NA | NA | TN | TN |
| Lead cyanamidate | 20837-86-9 | NA | NA | NA | NA | NA | TN | TN | TN | TN | NA | NA | TN | TN |
| Lead dinitrate | 10099-74-8 | NA | NA | NA | NA | NA | TN | TN | NA | NA | TN | NA | TN | TN |
| Lead monoxide (lead oxide) | 1317-36-8 | NA | NA | NA | NA | NA | TN | TN | NA | NA | FP | NA | FP | TN |
| Lead oxide sulfate | 12036-76-9 | NA | NA | NA | NA | NA | TN | TN | TN | NA | NA | NA | TN | TN |
| Lead titanium trioxide | 12060-00-3 | NA | NA | NA | NA | NA | TN | TN | TN | NA | NA | NA | TN | TN |
| Lead titanium zirconium oxide | 12626-81-2 | NA | NA | NA | NA | NA | TN | TN | TN | NA | NA | NA | TN | TN |
| Methoxyacetic acid | 625-45-6 | TN | TN | TN | NA | TN | TN | TN | TN | TN | FP | TN | TN | TN |
| Methyloxirane (Propylene oxide) | 75-56-9 | TN | TN | TN | FP | TN | TP | TP | TP | TP | TP | TP | TN | TP |
| N,N-dimethylformamide | 68-12-2 | TN | TN | TN | NA | TN | TN | TN | TN | TN | TN | TN | TP | TN |
| N-methylacetamide | 79-16-3 | TN | TN | TN | FP | TN | TN | TN | FP | FP | FP | TN | FP | TN |
| N-pentyl-isopentylphthalate | 776297-69-9 | FP | TN | FP | FP | FP | FP | TN | TN | TN | TN | TN | TN | TN |
| o-aminoazotoluene | 97-56-3 | TN | FP | TN | NA | TN | TP | FP | TP | FP | FP | FP | TP | FP |
| o-toluidine | 95-53-4 | TN | TN | TN | NA | TN | TP | FP | TP | FP | TP | FP | TP | FP |
| Orange lead (lead tetroxide) | 1314-41-6 | NA | NA | NA | NA | TN | TN | TN | NA | NA | NA | NA | TN | TN |
| Pentacosafluorotridecanoic acid | 72629-94-8 | FN | TP | FN | NA | FN | FP | TN | NA | TN | TN | TN | FP | TN |
| Pentalead tetraoxide sulphate | 12065-90-6 | NA | NA | NA | NA | NA | TN | TN | TN | NA | NA | NA | TN | TN |
| Pyrochlore, antimony lead yellow | 8012-00-8 | NA | NA | NA | NA | NA | TN | TN | TN | NA | NA | NA | TN | TN |
| Silicic acid (H2Si2O5), barium salt (1:1), lead-doped | 68784-75-8 | NA | NA | NA | NA | NA | TN | TN | TN | NA | NA | NA | TN | TN |
| Silicic acid, lead salt | 11120-22-2 | NA | NA | NA | NA | NA | TN | TN | TN | NA | NA | NA | TN | TN |
| Sulfurous acid, lead salt, dibasic | 62229-08-7 | NA | NA | NA | NA | NA | TN | TN | TN | NA | NA | NA | TN | TN |
| Tetraethyllead | 78-00-2 | NA | NA | NA | NA | NA | TN | TN | NA | NA | FP | NA | FP | TN |
| Tetralead trioxide sulphate | 12202-17-4 | NA | NA | NA | NA | NA | TN | TN | TN | NA | NA | NA | TN | TN |
| Tricosafluorododecanoic acid | 307-55-1 | TP | TP | FN | NA | TP | FP | TN | NA | TN | FP | TN | FP | TN |
| Trilead bis(carbonate) dihydroxide | 1319-46-6 | NA | NA | NA | NA | NA | TN | TN | TN | NA | NA | NA | TN | TN |
| Trilead dioxide phosphonate | 12141-20-7 | NA | NA | NA | NA | NA | TN | TN | TN | NA | NA | NA | TN | TN |
| 1,2-bis(2-methoxyethoxy)ethane (TEGDME,triglyme) | 112-49-2 | TN | TN | TN | NA | TN | TN | TN | FP | TN | FP | TN | FP | TN |
| 1,2-dimethoxyethane,ethylene glycol dimethyl ether (EGDME) | 110-71-4 | TN | TN | TN | NA | TN | TN | TN | FP | TN | TN | TN | TN | TN |
| 1,3,5-Tris(oxiran-2-ylmethyl)-1,3,5-triazinane-2,4,6-trione (TGIC) | 2451-62-9 | TN | FP | TN | FP | TN | FP | TP | FP | TP | TN | TP | FP | TP |
| 1,3,5-tris[(2S and 2R)-2,3-epoxypropyl]-1,3,5-triazine-2,4,6-(1H,3H,5H)-trione (β-TGIC) | 59653-74-6 | TN | FP | TN | FP | TN | FP | TP | FP | TP | TN | TP | FP | TP |
| 4,4'-bis(dimethylamino)-4''-(methylamino)trityl alcohol | 561-41-1 | TN | FP | TN | NA | TN | TP | FP | TP | FP | FN | FP | TP | FP |
| 4,4'-bis(dimethylamino)benzophenone (Michler’s ketone) | 90-94-8 | TN | FP | TN | NA | TN | TP | FP | TP | FP | TP | FP | TP | FPFP |
| [4-[4,4'-bis(dimethylamino) benzhydrylidene]cyclohexa-2,5-dien-1-ylidene]dimethylammonium chloride  (C.I. Basic Violet 3) | 548-62-9 | TN | FP | TN | NA | TN | TP | FP | TP | FP | FN | FP | TP | FP |
| [4-[[4-anilino-1-naphthyl][4-(dimethylamino)phenyl]methylene]cyclohexa-2,5-dien-1-ylidene] dimethylammonium chloride (C.I. Basic Blue 26) | 2580-56-5 | TN | FP | TN | NA | TN | TP | FP | TP | FP | FN | FP | TP | TN |
| Diboron trioxide | 1303-86-2 | NA | NA | NA | NA | NA | TN | TN | NA | NA | FP | TN | FP | TN |
| Formamide | 75-12-7 | TN | TN | TN | NA | TN | TN | TN | FP | TN | TN | TN | TN | TN |
| Lead(II) bis(methanesulfonate) | 17570-76-2 | NA | NA | NA | NA | NA | TN | TN | TN | NA | NA | NA | TN | FP |
| N,N,N',N'-tetramethyl-4,4'-methylenedianiline (Michler’s base) | 101-61-1 | TN | FP | TN | NA | TN | TP | FP | TP | FP | TP | FP | TP | FP |
| α,α-Bis[4-(dimethylamino)phenyl]-4 (phenylamino)naphthalene-1-methanol (C.I. Solvent Blue 4) | 6786-83-0 | FP | FP | FP | NA | FP | TP | FP | TP | FP | FN | FP | TP | FP |
| 1,2-dichloroethane | 107-06-2 | TN | FP | TN | FP | TN | TP | FP | TP | FP | TP | FP | TP | FP |
| 2,2'-dichloro-4,4'-methylenedianiline | 101-14-4 | TN | FP | TN | NA | TN | TP | FP | TP | FP | TP | FP | TP | FP |
| 2-Methoxyaniline, o-Anisidine | 90-04-0 | TN | FP | TN | NA | TN | TP | FP | TP | FP | TP | FP | TP | TN |
| 4-(1,1,3,3-tetramethylbutyl)phenol | 140-66-9 | TN | FP | FP | NA | TN | TN | TN | TN | TN | TN | TN | TN | TN |
| Arsenic acid | 7778-39-4 | NA | NA | NA | NA | NA | TP | TN | NA | NA | TP | TN | TP | TN |
| Bis(2-methoxyethyl) ether | 111-96-6 | TN | FP | TN | NA | TN | TN | TN | FP | TN | FP | TN | FP | TN |
| Bis(2-methoxyethyl) phthalate | 117-82-8 | TN | FP | TN | FP | TN | FP | TN | TN | TN | TN | TN | TN | TN |
| Calcium arsenate | 7778-44-1 | NA | NA | NA | NA | NA | TP | TN | FN | NA | NA | NA | TP | TN |
| Dichromium tris(chromate) | 24613-89-6 | NA | NA | NA | NA | NA | TP | TN | FN | NA | NA | NA | TP | TN |
| Formaldehyde, oligomeric reaction products with aniline | 25214-70-4 | TN | TN | TN | NA | TN | TP | FP | TP | FP | FN | FP | TP | FP |
| Lead diazide, Lead azide | 13424-46-9 | NA | NA | NA | NA | NA | FP | FP | TN | FP | NA | NA | FP | FP |
| Lead dipicrate | 6477-64-1 | NA | NA | NA | NA | NA | FP | FP | TN | FP | NA | NA | FP | FP |
| Lead styphnate | 15245-44-0 | NA | NA | NA | NA | NA | FP | FP | FP | FP | TN | TN | FP | FP |
| N,N-dimethylacetamide | 127-19-5 | TN | TN | TN | NA | TN | TN | TN | FP | FP | TN | TN | TN | TN |
| Pentazinc chromate octahydroxide | 49663-84-5 | NA | NA | NA | NA | NA | TP | TN | FN | NA | NA | NA | TP | TN |
| Phenolphthalein | 77-09-8 | TN | FP | TN | NA | TN | FN | TN | FN | TN | TP | TN | FN | TN |
| Potassium hydroxyoctaoxodizincatedichromate | 11103-86-9 | NA | NA | NA | NA | NA | TP | TN | FN | NA | NA | NA | TP | TN |
| Trilead diarsenate | 3687-31-8 | NA | NA | NA | NA | NA | TP | TN | FN | NA | NA | NA | TP | TN |
| 1,2,3-trichloropropane | 96-18-4 | TN | FP | TN | FP | TN | TP | TN | TP | FP | TP | FP | TP | FP |
| 1,2-Benzenedicarboxylic acid, di-C6-8-branched alkyl esters, C7-rich | 71888-89-6 | FP | TN | FP | FP | FP | FP | TN | TN | TN | FP | TN | FP | TN |
| 1,2-Benzenedicarboxylic acid, di-C7-11-branched and linear alkyl esters | 68515-42-4 | TN | TN | TN | TN | TN | FP | TN | FP | TN | FP | TN | FP | TN |
| 1-Methyl-2-pyrrolidone (NMP) | 872-50-4 | TN | TN | TN | NA | TN | TN | TN | TN | TN | TN | TN | TN | TN |
| 2-ethoxyethyl acetate | 111-15-9 | TN | TN | TN | FP | TN | TN | TN | TN | TN | TN | TN | TN | TN |
| Hydrazine | 302-01-2 | NA | NA | NA | NA | NA | TP | FP | Na | NA | FP | FP | TP | FP |
| Strontium chromate | 7789-06-2 | NA | NA | NA | NA | NA | TP | TN | NA | NA | NA | NA | TP | TN |
| 2-ethoxyethanol | 110-80-5 | TN | TN | TN | NA | TN | TN | TN | FP | TN | FP | TN | FP | TN |
| 2-methoxyethanol | 109-86-4 | TN | TN | TN | NA | TN | TN | TN | TN | TN | TN | TN | TN | TN |
| Dichromic acid | 7738-94-5 | NA | NA | NA | NA | NA | TP | TN | NA | NA | TP | TN | TP | TN |
| Chromium trioxide | 1333-82-0 | NA | NA | NA | NA | NA | TP | FN | NA | NA | TP | TP | TP | TP |
| Cobalt(II) carbonate | 513-79-1 | NA | NA | NA | NA | NA | TP | TN | NA | TN | FN | FP | TP | TN |
| Cobalt(II) diacetate | 71-48-7 | NA | NA | NA | NA | NA | TP | TN | FN | FP | FN | TN | FN | TN |
| Cobalt(II) dinitrate | 10141-05-6 | NA | NA | NA | NA | NA | TP | TN | NA | NA | TP | FP | TP | FP |
| Cobalt(II) sulphate | 10124-43-3 | NA | NA | NA | NA | NA | TP | TN | NA | FP | FN | FP | TP | FP |
| Ammonium dichromate | 7789-09-5 | NA | NA | NA | NA | NA | TP | FN | NA | NA | NA | NA | TP | FN |
| Boric acid | 10043-35-3 | NA | NA | NA | NA | NA | TN | TN | NA | NA | FP | TN | FP | TN |
| Disodium tetraborate, anhydrous | 1303-96-4 | NA | NA | NA | NA | NA | TN | TN | FN | NA | NA | NA | TN | TN |
| Potassium chromate | 7789-00-6 | NA | NA | NA | NA | NA | TP | FN | NA | NA | NA | NA | TP | FN |
| Potassium dichromate | 7778-50-9 | NA | NA | NA | NA | NA | TP | FN | NA | NA | NA | NA | TP | FN |
| Sodium chromate | 7775-11-3 | NA | NA | NA | NA | NA | TP | FN | FN | NA | NA | NA | TP | FN |
| Tetraboron disodium heptaoxide, hydrate | 12267-73-1 | NA | NA | NA | NA | NA | TN | TN | TN | NA | NA | NA | TN | FN |
| Trichloroethylene | 79-01-6 | TN | FP | TN | NA | TN | TP | FP | TP | FP | FN | FP | TP | FP |
| Acrylamide | 79-06-1 | TN | TN | TN | FP | TN | TP | TP | TP | TP | TP | TP | TP | TP |
| 2,4-dinitrotoluene | 121-14-2 | TN | FP | TN | NA | TN | TP | FP | TP | FP | TP | FP | TP | FP |
| Diisobutyl phthalate | 84-69-5 | TN | TN | TN | NA | TN | FP | TN | TN | TN | TN | TN | TN | TN |
| Lead chromate | 7758-97-6 | NA | NA | NA | NA | NA | TP | TN | FN | NA | NA | NA | TP | TN |
| Lead chromate molybdate sulphate red (C.I. Pigment Red 104) | 12656-85-8 | NA | NA | NA | NA | NA | TP | TN | FN | NA | NA | NA | TP | TN |
| Lead sulfochromate yellow  (C.I. Pigment Yellow 34) | 1344-37-2 | NA | NA | NA | NA | NA | TP | FP | FN | NA | NA | NA | TP | FP |
| Tris(2-chloroethyl) phosphate | 115-96-8 | TN | FP | TN | FP | TN | FP | FP | FP | FP | FP | FP | FP | FP |
| 4,4'- Diaminodiphenylmethane (MDA) | 101-77-9 | TN | FP | TN | NA | TN | TP | FP | TP | FP | TP | FP | TP | FP |
| 5-tert-butyl-2,4,6-trinitro-m-xylene (Musk xylene) | 81-15-2 | FN | TP | FN | NA | FN | TN | TN | TN | TN | TN | TN | TN | TN |
| Anthracene | 120-12-7 | FN | TP | FN | NA | FN | FP | FP | FP | TN | TN | FP | FP | FP |
| Benzyl butyl phthalate (BBP) | 85-68-7 | FP | TN | TN | FP | TN | FP | TN | TN | TN | FP | TN | FP | TN |
| Bis (2-ethylhexyl)phthalate (DEHP) | 117-81-7 | TN | TN | FP | TN | TN | FP | TN | TN | TN | FP | TN | FP | TN |
| Bis(tributyltin) oxide (TBTO) | 56-35-9 | NA | NA | NA | NA | NA | TN | TN | TN | TN | TN | TN | TN | TN |
| Cobalt dichloride | 7646-79-9 | NA | NA | NA | NA | NA | TP | TN | NA | NA | TP | FP | TP | FP |
| Diarsenic pentaoxide | 1303-28-2 | NA | NA | NA | NA | NA | TP | TN | NA | NA | FN | FP | TP | FP |
| Diarsenic trioxide | 1327-53-3 | NA | NA | NA | NA | NA | TP | TN | NA | NA | TP | FP | TP | FP |
| Dibutyl phthalate (DBP) | 84-74-2 | TN | TN | TN | TN | TN | FP | TN | TN | TN | TN | TN | TN | TN |
| Hexabromocyclododecane | 25637-99-4 | TP | TP | TP | TP | TP | FP | TN | FP | FP | TN | TN | FP | TN |
| Lead hydrogen arsenate | 7784-40-9 | NA | NA | NA | NA | NA | TP | TN | NA | NA | FP | NA | TP | TN |
| Sodium dichromate | 10588-01-9 | NA | NA | NA | NA | NA | TP | FN | FN | NA | NA | NA | TP | FN |
| Triethyl arsenate | 15606-95-8 | TN | FP | TN | NA | TN | TP | TN | TP | TN | TP | TN | TP | TN |
